# An overview of the anti-cancer actions of Tanshinones, derived from *Salvia miltiorrhiza* (Danshen)

**DOI:** 10.37349/etat.2020.00010

**Published:** 2020-06-29

**Authors:** Irum Naz, Myriam Merarchi, Shanaya Ramchandani, Muhammad Rashid Khan, Muhammad Nouman Malik, Sumaira Sarwar, Acharan S Narula, Kwang Seok Ahn

**Affiliations:** 1 Department of Biochemistry, Faculty of Biological Sciences, Quaid-i-Azam University, Islamabad 45320, Pakistan; 2 Faculty of Pharmacy, University of Paris Descartes, 75006 Paris, France; 3 Department of Pharmacology-Biomedicine, The University of Melbourne, Parkville, VIC 3010, Australia; 4 Higher Education Commission of Pakistan, Islamabad 44000, Pakistan; 5 Narula Research, Chapel Hill, NC 27516, USA; 6 Department of Science in Korean Medicine, College of Korean Medicine, Kyung Hee University, 24 Kyungheedae-ro, Dongdaemun-gu, Seoul 02447, South Korea; National University of Singapore, Singapore

**Keywords:** Tanshinone, cancer, signalling pathways, apoptosis, angiogenesis, pharmacokinetics

## Abstract

Tanshinone is a herbal medicinal compound described in Chinese medicine, extracted from the roots of *Salvia miltiorrhiza* (Danshen). This family of compounds, including Tanshinone IIA and Tanshinone I, have shown remarkable potential as anti-cancer molecules, especially against breast, cervical, colorectal, gastric, lung, and prostate cancer cell lines, as well as leukaemia, melanoma, and hepatocellular carcinoma among others. Recent data has indicated that Tanshinones can modulate multiple molecular pathways such as PI3K/Akt, MAPK and JAK/STAT3, and exert their pharmacological effects against different malignancies. In addition, preclinical and clinical data, together with the safety profile of Tanshinones, encourage further applications of these compounds in cancer therapeutics. In this review article, the effect of Tanshinones on different cancers, challenges in their pharmacological development, and opportunities to harness their clinical potential have been documented.

## Introduction

Cancer is the second leading cause of death worldwide and causes a significant loss of human life. In recent years, the incidence and mortality rates of cancer have shown rapid growth, thus, becoming a major public health challenge. According to a recent report published by the World Health Organisation, cancer is the cause of 70% of deaths in low and medium-income countries [1]. Currently, antineoplastic drugs being used for the treatment of cancer can be divided into two categories: targeted and non-targeted drugs. Non-targeted drugs such as Adriamycin, Cisplatin, and Paclitaxel can effectively inhibit tumor progression but have shown severe side effects on human normal cells [2]. Targeted drugs are available in the market that can affect specific oncogenic signalling pathways; however, high cost makes these drugs unaffordable in many developing countries. Hence, the discovery of low-cost targeted drugs has become an urgent need for cancer therapy. For millennia, natural products, including those derived from plants, animals, and microorganisms, have been extracted and synthesized [3, 4]. These products can provide prototypes for the synthesis of pharmacologically active agents, specifically, antineoplastic agents [5–9] and have been found to be effective against different cancers [10–13]. This review analyzes the significance of Tanshinones in cancer prevention as well as treatment in different tumor models.

Tanshinone (Tan) is a herbal medicinal compound reported in Chinese medicine. More than forty Tan compounds have been extracted from *Salvia miltiorrhiza* (Danshen), although some compounds have also been derived from other plant species including *S. argentea*, *S. aerea*, *S. kiaometiensis*, *S. vasta*, and *S. yunnanensis* [14]. Interestingly, Tan compounds identified in *Salvia miltiorrhiza* roots (Formula: C_19_H_18_O_3_) are liposoluble naphthoquinone diterpenes and have been reported to exhibit great potential against cardiovascular diseases and for stroke treatment. Tanshinones, including Tanshinone I (Tan I) and Tanshinone IIA (Tan IIA) can suppress the growth of multiple cancers including those of bladder [15], breast [16–18], cervical [19], liver [20, 21], lung [2–31], pancreatic and prostate cancer (PC) [32–36]. In addition, these bio-active compounds can modulate the growth of astrocytoma [37], endometrial carcinoma [38], mesothelioma [39], melanoma, and leukaemia [40, 41] as well as osteosarcoma [42] and ovarian carcinoma [43–45].

**Figure 1.**
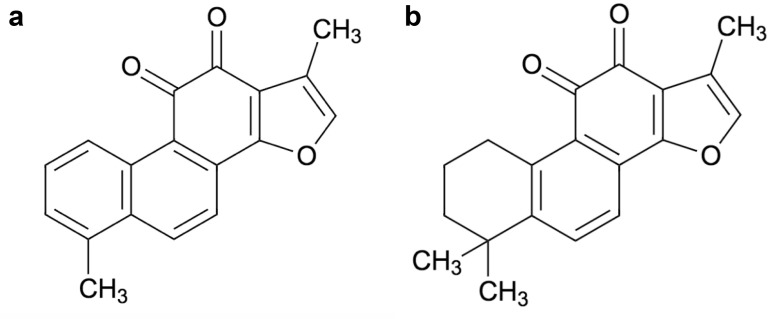
Molecular structure of Tanshinones: (a) Tan I and (b) Tan IIA

Tan I compound (C_19_H_18_O_3_) has a molecular weight of 276.3 g/mol (Figure 1a), and has been identified from *Salvia* species. Tan I can display extensive anticoagulant, immunosuppressive, antineoplastic, phytogenic, anti-inflammatory, and anti-infective properties. Among Tanshinones, Tan IIA (molecular weight of 294.3 g/mol) is the most abundant compound from the *Salvia* species and its structure has been shown in Figure 1b. Interestingly, Tan IIA, in addition to its promising antineoplastic effects, can also exhibit anti-inflammatory, non-steroidal, and anti-infective properties. In general, Tanshinones have poor bioavailability and can be more effective when injected intravenously. This article aims to provide an updated review of Tan compounds and their modes of actions against different malignancies.

## Molecular pathways affected by Tan

The various Tan compounds have the ability to target multiple oncogenic signalling pathways and abrogate malignant transformation. Numerous studies have been conducted on Tan IIA and Tan I compounds to determine their effect on various pathways that can promote tumorigenesis and these are discussed briefly below.

### Tan IIA

**Figure 2.**
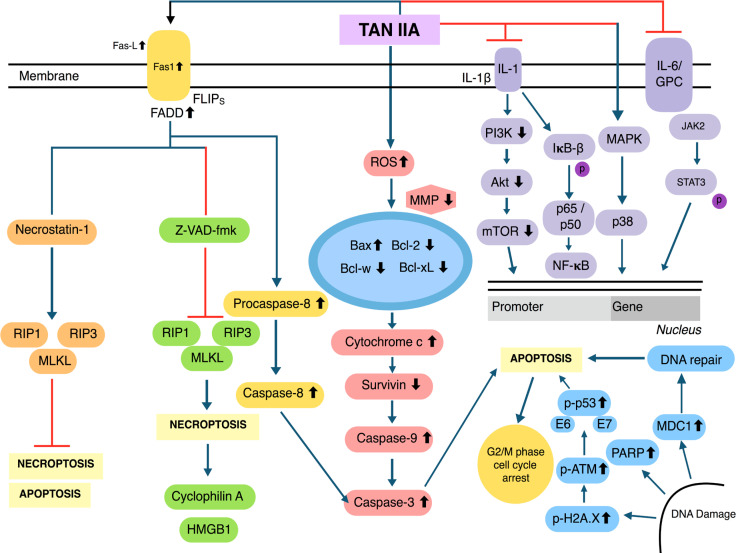
Multiple molecular signalling pathways of Tan IIA. MMP: matrix metalloproteinase; HMGB1: high-mobility group protein B1

The generation of reactive oxygen species (ROS) inside mitochondria can promote oxidative stress which can regulate the activation of apoptotic signalling pathways [46–49]. Upon treatment with Tan IIA, the level of ROS was elevated, resulting in inductionof apoptosis through the modulation of phosphoinositide 3-kinase (PI3K), AKT, m-TOR, and c-Jun N-terminal kinase (JNK) pathways [43, 50, 51]. Tan IIA has been found to suppress the adenosine monophosphate-activated protein kinase (AMPK) pathway resulting in the inactivation of Sphase kinase associated protein 2 (Skp2), which can promote mitochondrial apoptosis via inhibition of mitophagy [52]. Human papillomavirus (HPV), a well-known causative agent of cervical cancer, encodes the E6 and E7 viral oncoprotein, which can result in the inactivation of tumor suppressor proteins p53 and retinoblastoma protein (pRB). In HPV positive human cervical cancer CaSki cells, Tan IIA displayed multiple pharmacological effects and reduced the expression of E6 and E7 and eventually altered the levels of proteins such as E6AP, E2F1, and pRb. It also triggered p53 accumulation, altered p53-dependent expression of target genes, and mediated p53 dependent apoptosis through modulation of B-cell lymphoma-2 (Bcl-2), caspase-3, and poly ADP-ribose polymerase (PARP) expression [19]. Interestingly, Tan IIA also caused both Nec-1 inhibition and FLICE inhibitory protein in short form (FLIP_s_) mediated apoptosis/necroptosis [53]. Moreover, Tan IIA can affect multiple transcription factors and modulate the levels of phosphorylated p65, and in turn, suppressed the activation of nuclear factor kappa light chain enhancer of activated B cells (NF-κB) signalling pathway and also abrogate JAK/STAT3 signalling pathway [54, 55]. Through these molecular mechanisms, Tan IIA may have the ability to target specific proteins in human cancer cells. The multiple molecular mechanisms of action of Tan IIA have been summarized below (Figure 2).

### Tan I

**Figure 3.**
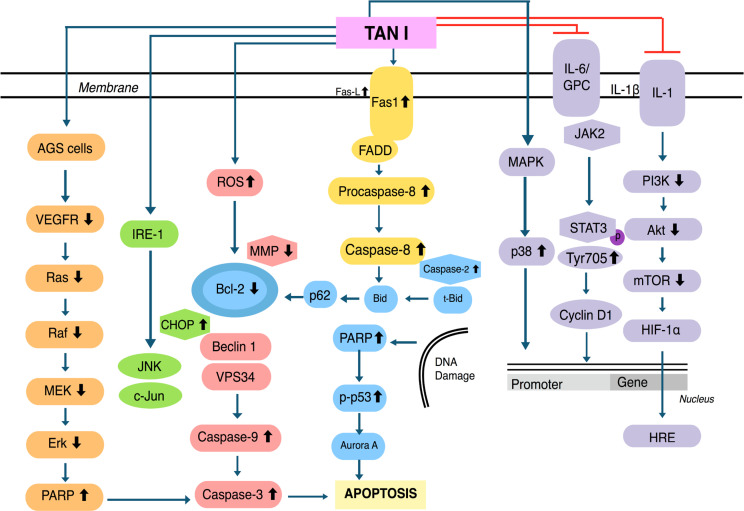
Multiple molecular signalling pathways regulated by Tan I

Pre-treatment with Tan I on various malignancies induced apoptosis through an increased ROS production and substantially reduced the MMPs level [38]. Additional studies have indicated that Tan I can induce apoptosis and autophagy through the aggregation of p62 (C-terminal UBA domain being required for this aggregation), as well as by causing an up-regulation of inositol-requiring protein-1 (IRE1), CCAAT-enhancer-binding protein homologous protein (CHOP), and p-c-Jun N-terminal kinase (p-JNK) [39]. In addition, Tan I was found to reduce Bcl-2 protein expression, as well as increase the conversion of LC3I to LC3II and trigger autophagosome formation without altering the expression of Beclin-1. This could increase Beclin1-VPS_34_ complexes and thus induce both apoptosis as well as autophagy [56]. Lu et al. [57], documented that Tan I affected Aurora A-p53 and survivin-induced signalling pathways and caused an upregulation of PARP levels, which enhanced p-p53 protein expression and reduced the levels of Aurora A kinase. This action, as well as the modulation of caspase-3 and caspase-9 led to an induction of apoptosis in colorectal cancer (CRC) cells. In addition, Tan I cleaved procaspase-8, which attenuated the expression of Bid and t-Bid, thereby in turn activating caspase-2. In contrast, Tan IIA has been shown to modulate vascular epidermal growth factor receptor (VEGFR) as well as Ras/Raf/MEK/ERK pathway in AGS gastric carcinoma cells [58]. Moreover, Tan I downregulated the activation of the PI3K/Akt/m-TOR pathway leading to a substantial level of apoptosis in breast cancer cells [17]. The molecular mechanisms of Tan I have been briefly depicted in the figure below (Figure 3).

## Anti-cancer effects of Tan

Despite the use of Tan compounds to treat cardiovascular disease and strokes in Traditional Chinese Medicine, they also have exhibited antineoplastic effects against numerous malignancies. This section of the review will further elaborate on these anti-tumoral effects, including the induction of apoptosis, inhibition of angiogenesis, and metastasis against different tumor cells (Table 1).

**Table 1.** Selected anti-cancer effects of Tan compounds in-vitro

**Cancer type**	**Cell line**	**Pathways/molecules altered**	**References**
Astrocytoma	Astrocytoma cells	↑Notch-1	[97]
Breast cancer	MDA-MB-231, MCF-7	↓HIF-1α, ↓VEGF	[102] [16]
Bladder cancer	BCa	↓EMT	[15]
Cervical cancer	HPV positive CaSki	↓E6 and E7, ↑p53	[19]
		↓CYP2J2-mediated astemizole O-demethylation	[19] [117]
Cervix carcinoma	Stemness-like cancer cells	↓YAP mRNAs	[98]
Colon cancer	SW480	↑E-cadherin, ↓vimentin and MMP-9	[118]
	CRC	↓AMPK, ↑Skp2	[52]
Esophageal cancer	EC-109	↑caspase-4, ↑CHO	[24]
Gastric cancer	AGS	↓EGFR, ↓IGFR, ↓PI3K, ↓AKT, ↓mTOR, ↓NF-κB-p65, ↓COX-2 and MMP-2, -7 and -9 S, ↑p-p38, ↑p-JNK, ↑p53, ↑p21, ↑caspase-3 and caspase-8, ↑PARP ↓VEGFR, ↓HER2, ↓Ras, ↓Raf, ↓MEK, ↓ERK	[119] [58]
Gastric cancer	SGC-7901	↓Ki-67, ↓PCAN, ↓MMP-2, ↓MMP-9, ↓FOXM1, ↑P21	[69]
	SNU-638 & MKN1	↑Bcl-2, ↓caspase-3, ↓p-STAT3	[54] [37]
Glioma	U251	↓p-PI3K, ↓Bcl-2, ↓p-Akt, ↑Bax, ↑LC3B, ↑Beclin 1	[37]
Leukaemia	APL	C/EBPβ	[99]
Liver cancer	HepG2	↓CYP2J2 activity	[117]
	HepG2	↑LDLR, ↑p53, ↓HNF-1α, ↓Nec-1, ↓PCSK9	[53] [81]
Lung cancer	A549	↑Cleaved Caspase-3 and Bax, ↓VEGF, ↓VEGFR2, ↓p-PI3K, ↓p-Akt, ↓Bcl-2, ↓Caspase-3 protein	[25] [26] [30] [31]
Melanoma	A375	↑Beclin-1, ↑LC3-II expression, ↓pPI3K, ↓p-AKT, ↓p-mTOR, ↓p-p7036k1	[41]
Oral cancer	SCC-9 squamous carcinoma	↑ROS, ↑Beclin 1, ↑Atg5	[89] [90]
Ovarian cancer	TOV-21G	↑TRAIL-induced apoptosis, ↑DR5, ↑ROS-JNK-CHOP, ↑miR-205, ↑Bcl-2 ↓Mcl-1L, ↓PI3K, ↓AKT, ↓JNK	[43] [45]
Osteosarcoma	143B	↓Ki67, ↓PCNA	[42]
	MG-63	↑Cleaved-PARP, ↑ROS, ↑Caspase-3, -8 and -9	[96]
PC	PC-3	↓Beclin1, ↓LC3-II	[32]
	LNCaP	↑maspin, ↑ARs	[120]
	PC12	↓p-Akt, ↓p-ERK1/2, ↑p-FOXO3a, ↑c-Raf	[121]

EMT: epithelial-mesenchymal transition; FoxM1: forkhead box M1; MMP-2: matrix metalloproteinase 2; MMP-9: matrix metalloproteinase 9

### Effect of Tanshinones against different cancers

#### Tan and breast cancer

According to the World Health Organisation, breast cancer is the second-most commonly occurring cancer worldwide, following lung cancer [59–62]. Like many other cancers, chemoresistance remains a notable issue when using Taxol; a well-known chemotherapeutic agent used in breast cancer patients. Tan IIA in combination with Taxol was found to considerably reduce the resistance of breast cancer MCF-7 (taxol resistant) cells by causing an abrogation of microtubule associated protein [16]. It is interesting to note that both estrogen receptor-responsive MCF-7 and estrogen-independent MDA-MB-453 human breast cancer cells were found responsive to Tan I, irrespective of their sensitivity to estrogen [17]. In addition, Tan I attenuated the levels of HIF-1α and p-705-STAT3 and the secretion of VEGF in MCF-7 cells [63]. Likewise, an acetyl Tan IIA, a chemically modified Tan IIA derivative can induce apoptosis through increased ROS generation, by increasing the level of pro-apoptotic Bcl-2-associated-X protein (Bax), thereby causing caspase-3 activation and cytochrome c release in HER2 positive breast cancer cells [18].

#### Tan and cervical cancer

According to the American Institute of cancer research, cervical cancer is the fourth-deadliest cancer in women and ranks eighth in most commonly occurring cancer worldwide [64]. Cervical cancer interventions mainly focus on primary and secondary prevention strategies. Primary prevention is the best way to diminish the burden of cervical cancer and its related mortality. HPV is recognized as an etiological factor in cervical cancer induction [65]. In HPV, the oncogenes *E6* and *E7* have been reported to inactivate tumor suppressor proteins p53 and pRb. In HPV positive CaSki cells, it has been shown that Tan IIA can down-regulate the expression of these vital oncogenes by regulating the expression of their associated proteins E6AP, E2F1, pRb that can cause an accumulation of p53 and p53-dependent downstream targets and can also modulate the levels of Bcl-2, Bax, and caspase-3 thereby promoting PARP cleavage [19]. Furthermore, in HPV positive CaSki cells, Tan IIA can induce apoptosis and block the S phase progression of the cell cycle effectively [19].

#### Tan and colorectal & gastric cancer

Following lung and breast cancer, CRC is the third-deadliest cancer, particularly in Western countries, whereas chemoresistance is a major problem in gastric cancer [1, 66]. It is, therefore, imperative to find suitable treatments for these cancers. In Tan I-treated HCT116 and SW480 human CRC cells, inhibition of cell proliferation along with a decreased level of cyclin D1 protein was observed [67]. In addition, in CRC cells, Tan I induced apoptosis and specifically suppressed CRC cell growth [67]. A study reported by Kim et al. [68], has indicated a significant potential of Tan I in reducing the viability of HCT116 and HT29 cells. On the contrary, Tan IIA was shown to enhance CRC apoptosis through the suppression of the MAPK pathway and the inactivation of Spk2 proteins [52]. A study reported by Jing et al. [56], indicated that Tan I could effectively suppress the proliferation, as well as promote apoptosis in BGC823, and SGC7901 human gastric cancer cells. Likewise, Tan IIA was noted to significantly induce apoptosis, inhibit migration and proliferation in AGS and SGC-7901 gastric cancer cells [58, 69]. Tan IIA effectively suppressed the proliferation of multiple gastric cancer cell lines including SNU-638, MKN1, and AGS, while inducing apoptosis via mediating increased level of cleaved caspase-3 and decreased expression of Bcl-2 [54]. It is important to note that Dihydrotanshinone (DT), another derivative of Tanshinones, displayed significantly higher cytotoxicity when compared with other Tanshinones in SGC7901 and MGC803 gastric cancer cells [70]. However, exposure of Tan IIA on colon cancer cell lines including SW480 and HC8693 reduced cellular growth, and invasive potential [71]. In addition, IL-2-based treatment in combination with Tan IIA enhanced the IL-2-mediated cell death and reduced proliferation of SW480 cells [72]. In another study by using similar SW837 cells, Tan IIA promoted cell death, impaired cell migration, and mediated proliferation arrest [73]. Overall, through these pleiotropic mechanisms, Tan I and Tan IIA compounds may have a substantial ability to inhibit the growth of colorectal and gastric cancers.

#### Tan and esophageal cancer

Esophageal cancer is one of the least curable cancers with a survival rate between 5–30% post five years of diagnosis [74]. Tan IIA can inhibit proliferation via modulating Akt pathway and directly induce cell cycle arrest by sequestering EC109 esophageal cancer cells in S phase [23]. Additionally, Tan IIA could also initiate apoptosis either through activation of CHOP/caspase pathway by sequentially increasing caspase-3 and caspase-9 expression, altering Bax/Bcl-2 ratio, or modulating endoplasmic reticulum caspase-4 and CHOP levels in a dose dependent manner [24]. Another study reported that Tan IIA can substantially suppress proliferation through targeting Cyclin B1 protein, which led to an interruption of cell cycle in S and G2/M phases and induced apoptosis in EC-1 and ECa-109 esophageal carcinoma cell lines [28].

#### Tan and hepatocellular carcinoma (HCC)

Liver cancer is the fifth most common cause of cancer death worldwide, whereas estimated deaths will rapidly keep on increasing in 2020 and beyond [75–79]. Tan IIA coupled with mPEG-PLGA-PLL-cRGD (methoxy polyethylene glycol, polylactic-co-glycolic acid, poly-L-lysine, and cyclic arginine-glycine-aspartic acid) when used to treat HCC has shown improved anti-tumoral activities [21]. Tan IIA in combination with trans-resveratrol exerted synergistic apoptosis and cytotoxicity comparable to that of cisplatin [20]. In HepG2 human liver cancer cells, Tan IIA upregulated low-density lipoprotein receptor (LDLR) and altered the levels of PCSK9 protein. Tan IIA also increased forkhead box class O 3a (FOXO3a) expression, which attenuated levels of PCSK9 protein in HepG2 cells [80]. Tan IIA modified apoptosis to necroptosis through promoting necrostatin-1 inhibition and downregulating FLIP_s_ receptor [53]. A study conducted by Ren et al. [81], stated that Tan IIA can exhibit anti-oncogenic activity through apoptosis induction caused by increased Bax/Bcl-2 ratio and altered levels of caspase-3, p21, cyclin D1, and cyclin dependent kinase 6 in hepatocellular carcinoma cells. Moreover, Tan IIA treatment upregulated the level of p53 protein as well as *PTPN11* and its encoded protein SHP2 that can mediate its possible anticancer activities [81].

#### Tan and lung cancer

Lung cancer is the leading cause of cancer-related death worldwide [82–86]. It was reported that Tan IIA suppressed proliferation, angiogenesis, activated apoptosis, and caused cell cycle arrest at the S phase through a decrease in the expression of vascular endothelial growth factor (VEGF) and VEGFR2 proteins in A549, PC9, and HLF lung cancer cells [25]. Additionally, Tan IIA in combination with adriamycin remarkably suppressed migration, activated apoptosis, and arrested cell cycle, particularly in A549 cells [26]. In radioresistant lung cancer H358-IR and H157-IR cells, treatment with Tan I caused an inhibition of cell proliferation, which could lead to an enhancement of radiosensitivity. Tan I was docked into the active site of phosphoribosyl pyrophosphate amidotransferase (PPAT) protein, and was found to attenuate PPAT signalling [29]. Likewise, in lung adenocarcinoma PC9 cells, DT arrested proliferation and activated the apoptotic process. The expression of Bax, IRE-1, Bip, and caspase-12 was augmented upon DT exposure and caused apoptosis [30]. Interestingly, Tanshinones could also attenuate non-small cell lung cancer growth by suppressing *AURKA* an oncogene via augmenting the expression of miR-32 and other related miRNAs [31], and thus can act as effective therapeutic agents.

#### Tan and oral cancer

Treatment for oral cancer thus far has not been quite promising when the tumor is detected at a metastatic stage, with a 5-year survival rate of around 39% [75, 87, 88]. In human oral squamous cell carcinoma (SCC09), Tan IIA can exhibit radio-sensitizing effects and can induce autophagy. Pre-treatment with Tan increased ROS generation, upregulated Beclin-1, Atg5, and LC3-III proteins [89]. In another study, the administration of Tan in SCC-9 cells increased apoptosis by modulating the PI3K/Akt pathway, increased cleaved-caspase-3 expression, as well as the ratio of LC3-II to LC3-I [90].

#### Tan and ovarian cancer

Ovarian cancer is the second most common cause of gynaecological cancer-associated mortality globally [75]. In a study conducted on ovarian cancer cells, Tan IIA caused activation of caspase -3, -8, and -9 and decreased the expression of Bcl-2 [43]. In A2780 human ovarian carcinoma cells, it has been demonstrated that Tan IIA can enhance TRAIL-induced apoptosis by up-regulating death receptor 5 (DR5) through the ROS/JNK/CHOP pathway. Indeed, DR5 was noted to be stimulated via the up-regulation of CCAAT/enhancer-binding protein homologous protein [44]. In ovarian carcinoma Tan IIA-treated TOV-21G cells, apoptosis was induced through the downregulation of survivin, while the levels of Bax, Bcl-2, and B-cell lymphoma-extra-large (Bcl-xL) remained unaffected. It also caused miR-205 overexpression, which altered the levels of survivin protein [45]. Specifically, through the modulation of ROS/JNK/CHOP pathway, Tan IIA can inhibit growth and induce apoptosis in ovarian cancer cell lines.

#### Tan and PC

PC is a leading cause of death in men, especially among the aged men [91–94]. Tan IIA-treated human PC-3 cells, displayed mitochondrial-dependent apoptosis and autophagy, that were found to be dependent on ROS production [32–34]. Moreover, PTS33, a new sodium derivative of cryptotanshinone was noted to selectively inhibit androgen receptor (AR) activities, and effectively suppress the growth of AR-positive PC cells, while the effect on AR-negative PC cells was limited [35]. The insulin-like growth factor 1 (IGF-1) and its receptor (IGF-1R) can facilitate tumor proliferation and progression. In another study, PC12 cells were treated with IGF-1 with or without Tan IIA, and the results have shown that IGF-1 can promote the growth of PC12 cells and Tan IIA inhibited their growth significantly [36].

#### Tan and other cancers

Tan compounds are able to target several other cancer cells, including bladder cancer, pancreatic cancer, osteosarcoma, melanoma, and leukaemia; the specific actions of Tanshinones on various cancers have been elaborated below. In human bladder cancer cell lines, 5637, BFTC and T24, Tan IIA was found to suppress the proliferation, migration, and metastasis [15]. It can abrogate the growth of MiaPaCa-2 pancreatic cancer cells by interrupting the ras/Raf/MEK/ERK and PI3K/AKT/mTOR pathways [95]. Tan IIA treated MG63 osteosarcoma cells exhibited reduced proliferation and autophagy induction [96]. Administration of Tan IIA to non-obese diabetic-severe combined immunodeficiency (SCID) mice implanted with human osteosarcoma 143B cells led to a significant inhibition of tumor development [42]. Malignant astrocytoma is the most common malignant tumor with strong invasion capability in the central nervous system, where Tan IIA had shown a significant anti-proliferative and pro-apoptotic effect in these cells [97]. In human glioma cell U251, Tan IIA was shown to inhibit the viability of the cells by inducing cells apoptosis and autophagy [37]. Additionally, in cervix carcinoma (CC) stem cells, it has been found that Tan IIA can suppress CC stemness-like cells migration and invasion [98]. In a study conducted on CC stemness-like cells, pre-treatment with Tan IIA was found to reduce migration that resulted in suppression of HuR protein translocation from the nucleus to the cytoplasm, thereby reducing YAP mRNA stability and transcriptional activity [98]. Tan I treated H28 and H2452 mesothelioma cells displayed high cytotoxicity along with autophagic features [39]. Furthermore, the effects of Tan IIA on melanoma A375, MV3, M14, and other human cell lines including HaCat and HUVEC cells were investigated and it has been discovered that Tan IIA inhibited melanoma A375, MV3, and M14 proliferation and reduced CXCL12 levels, in A375 cells, leading to a decrease in invasiveness and migration [41]. Finally, in Tan IIA-treated K562 cells (myelogenous leukaemia), a significant inhibitory effect on the growth, in addition to the activation of apoptosis was observed [99].

### *In vivo* effects of Tanshinones

The effects of Tan IIA have been evaluated pre-clinically in mouse models of numerous types of cancers. In nude mice bearing BGC823 xenograft (gastric cancer), it has been shown that the combination of chloroquine and Tan I could inhibit tumor growth more efficiently than monotherapy [56]. Tan IIA also suppressed cancer-induced bone pain, or cancer induced bone pain, which is a chronic condition as identified by both ongoing and breakthrough pain. Tan IIA attenuated the expression levels of spinal HMGB1 and levels of inflammatory markers such as IL-1β, tumor necrosis factor alpha (TNF-α), and IL-6 [100]. Moreover, Tan IIA significantly inhibited the neuronal responses of wide dynamic range neurons in spinal deep layers [100]. In Tan IIA treated human osteosarcoma cells 143B, tumor development was significantly inhibited, associated with attenuation of proliferation, migration and invasion, and apoptosis induction [42].

Tan-IIA also increased the sensitivity to irradiation in laryngeal cancer cells both *in vitro* and *in vivo* [27]. Tan IIA treatment led to a dramatic 66% reduction in tumor volume of cervical cancer xenograft athymic nude mice by lowering the expression of proliferation marker; proliferating cell nuclear antigen (PCNA) [19]. In addition, treatment of Tan IIA significantly abrogated the growth in ovarian tumor cells through inducing apoptosis [43]. *In vivo*, diterpenoid Tan promoted lung PC9 cell apoptosis in a dose-dependent manner, up-regulated Bip, IRE1, and TRAF2 protein expressions in tumor tissues, reduced tumor weight and alleviated weight loss [30]. In gastric cancer AGS cells, xenograft SCID mice were treated with Tan IIA for 8 weeks, which significantly decreased the expression levels of epidermal growth factor receptor (EGFR), IGFR, PI3K, AKT, and mTOR proteins in dose-dependent fashion [101]. In BxPC3 (pancreatic cancer) derived xenograft tumor, Tan IIA significantly suppressed the growth of the tumor. Human oral squamous cell carcinoma (OSCC) SCC-9 cells were injected in OSCC xenograft mice models and treated with Tan IIA. The consequent results showed an autophagy-inducing effect against OSCC in a Beclin-1-dependent mechanism of action [90]. In Tan IIA treated APL-bearing mice, a decrease in proliferation and apoptosis induction were observed along with a prolonged survival rate [99]. It has been found that Tan IIA inhibited HIF-1α and VEGF levels in human breast cancer xenografts, resulting in decreased angiogenesis [102]. Recent reports have shown that bone marrow-derived endothelial progenitor cells (EPCs) are potent regulators of angiogenesis, postnatal neovascularization, and metastasis. Tan IIA treatment led to the suppression of VEGF promoted migration and tube formation of human EPCs, without cytotoxic effects. Tan IIA had inhibited VEGF-induced angiogenesis in chick embryo chorioallantoic membrane models. There was also reduced microvessel formation and the expression of EPC- specific markers in mice [103] . Additionally, pre-treatment of ovarian cancer cells with Tan IIA resulted in increased apoptosis [43]. Acetyl Tan IIA derived from the chemical modification of Tan IIA inhibited tumor growth, induced apoptosis, increased water solubility and apoptotic activity on a greater number of cancer cell lines than Tan IIA [18]. ATA was also able to inhibit the growth of HER2 breast cancer positive cells injected in xenograft mice at a faster rate than those pre-treated with Tan IIA. Hence, these *in vivo* studies have revealed that Tan IIA can act as an effective anti-cancer drug. In addition, the beneficial effects of Tan compounds have been reported clinically on patients with polycystic ovary syndrome, APL and other cancers [104–112].

## Effect of Tan on angiogenesis

Numerous reports have depicted that anti-angiogenic potential of Tan compounds [102, 103]. Tan I can effectively reduce tumor angiogenesis by decreasing phosphorylation of STAT3 at tyrosine 705 residue. Tan I also inhibited angiogenesis in endothelial and tumor cells by reducing hypoxia-induced HIF-1α accumulation [63]. Moreover, Tan IIA can also attenuate β-catenin/VEGF-driven angiogenesis by affecting TGF-β1 in normoxic and HIF-1α in hypoxic microenvironments in colorectal cancer [113].

## Application of Tan in combination with nanoparticles

Significant efforts have been made to improve pharmacokinetic parameters of Tanshinones through novel formulations, including using solid lipid nanoparticles (SLN) combined with the Tan active compounds. SLN can protect the pharmaceutical compounds from degrading in various *in vivo* compartments in which they are released and favour a better control over the drug release profile [14]. In rats, it has been shown that Tan IIA-loaded SLN coated with poloxamer 188 can extend the time of plasma elimination and the mean residence time of Tan IIA [114], reduced opsonisation by serum proteins and macrophage uptake, as well as improved the circulation lifetimes for Tan IIA in plasma [115]. Another combination form that consisted of polylactic acid nanoparticles containing Tan IIA exhibited better performance in displaying pharmaceutical effects [116].

## Pharmacokinetic parameters of Tanshinones

It has been well established that Tanshinones can exhibit remarkable anticancer effects [117–121] but have a limited bioavailability when administered orally [14]. The distribution of four compounds cryptotanshinone, Tan IIA, danshensu, and ferulic acid from an extract was around 60% in rabbit plasma with a precision of 10%. Previous studies have indicated that the distribution and elimination of cryptotanshinones in rabbits are fast, however, together with danxiongfang, a compound used for the preparation of herbal medicine, it was shown to hinder its distribution being injected through intravenous route from a single dose. Cryptotanshinones at a dose of 100 mg/kg through oral administration and intraperitoneal routes in rat plasma have shown a bioavailability of 2.05% and 10.60%, respectively. Numerous research groups have measured diverse pharmacokinetic parameters of Tan compounds in various animal models [122–125] and their findings have aided in understanding the behaviour of these compounds inside the different organisms.

## Conclusions

Compounds extracted from natural agents such as medicinal plant sources possess numerous advantages including low cytotoxicity, capability to affect various oncogenic pathways and novel bioactive structures. They are widely studied due to their therapeutic potential and lower toxicity. A number of studies have suggested that Tanshinones, derived from *Salvia miltiorrhiza* roots possess remarkable potential as antineoplastic drugs. This review analyses the role of various Tanshinones in modulating multiple oncogenic factors that facilitate tumor formation, progression, and metastasis. It has been established that the majority of cancers arise due to a combination of inflammatory events, bacterial infections, dysfunctional cell death mechanisms, and deregulation of cell cycle molecules. Thus, Tanshinones that can target multiple hallmarks are good candidates to prevent/treat tumorigenesis. Interestingly, few reported clinical trials have shown promising results in different cancer patients. Nevertheless, it has been found in several studies that Tanshinones may have a limited bioavailability when administered orally, and thus efforts are being channelized to improve their pharmacokinetic parameters through developing novel formulations. Consequently, in the future, clinical trials will be primarily focused on large, randomized patient groups to better establish the anticancer potential of Tanshinones.

## Abbreviations

APL: acute promyelocytic leukaemia
AR: androgen receptor
As_4_S_4_: tetra-arsenic tetra-sulfide
ATRA: all-trans-retinoic acid
Bax: BCL2-associated X protein
Bcl-xL: B-cell lymphoma-extra large
CHOP: CCAAT-enhancer-binding protein homologous protein
CRC: colorectal cancer
DT: Dihydrotanshinone
EGFR: epidermal cell growth factor receptor
EMT: epithelial-mesenchymal transition
EPC: endothelial progenitor cell
EPCs: endothelial progenitor cells
FLIP_s_: FLICE inhibitory protein
FoxM1: forkhead box M1
HCC: hepatocellular carcinoma
HMGB1: high-mobility group protein B1
HIF1-α: hypoxia-inducible factor 1-alpha
HPV: human papillomavirus
HRE: hypoxia response element
IFGR: insulin-like growth factors
JNK: Jun N-terminal kinase
LDLR: low-density lipoprotein receptor
MAPK: monophosphate-activated protein kinase
MMP: matrix metalloproteinase
OSCC: oral squamous cell carcinoma
PARP: poly ADP-ribose polymerase
PC: prostate cancer
PCNA: proliferating cell nuclear antigen
PI3K: phosphoinositide 3-kinase
p-JNK: p-c-Jun N-terminal kinase
PPAT: phosphoribosyl pyrophosphate amidotransferase
Prb: retinoblastoma protein
ROS: reactive oxygen species
SCID: severe combined immunodeficiency
SLN: solid lipid nanoparticles
Tan: Tanshinone
Tan I: Tanshinone I
Tan IIA: Tanshinone IIA
VEGF: vascular endothelial growth factor
VEGFR: vascular epidermal growth factor receptor
Bcl-2: B-cell lymphoma-2
IRE1: inositol-requiring protein-1
IGF-1: insulin-like growth factor 1
IGF-1R: insulin-like growth factor type 1 receptor
IL-6: interleukin 6
MMP-2: matrix metalloproteinase 2
MMP-9: matrix metalloproteinase 9
HER2: human epidermal growth factor receptor 2
FOXO3a: forkhead box class O 3a
DR5: death receptor 5

## Declarations

### Copyright

© The Author(s) 2020.
